# CYP2C19 intermediate and poor metabolizer statuses may be associated with coronary atherosclerosis among patients with type 2 diabetes mellitus

**DOI:** 10.3389/fmed.2026.1738919

**Published:** 2026-01-22

**Authors:** Nan Cai, Youqian Li, Jingfeng Liu, Changjing Huang, Haifeng Hong, Hanlin Li, Junyin Peng

**Affiliations:** Center for Cardiovascular Diseases, Meizhou People's Hospital, Meizhou Academy of Medical Sciences, Meizhou, China

**Keywords:** coronary atherosclerosis, CYP2C19, dyslipidemia, susceptibility, type 2 diabetes mellitus

## Abstract

Type 2 diabetes mellitus (T2DM) has a higher risk of coronary atherosclerosis (CAS) compared to the general population. Cytochrome P450 2C19 (CYP2C19) closely related to the occurrence and development of cardiovascular diseases. This study intends to conduct research on the relationship between *CYP2C19* polymorphisms ((rs4244285, 681G > A, *2) and (rs4986893, 636G > A, *3)) and the risk of CAS in patients with T2DM. 4,627 T2DM patients from January 2019 to March 2024 were retrospectively analyzed, included 2,390 cases of T2DM complicated with CAS and 2,237 cases of patients with T2DM only. CYP2C19 was divided into three phenotypes: extensive metabolizer (EM), intermediate metabolizer (IM), and poor metabolizer (PM) based on rs4244285 and rs4986893 SNPs. The relationship between *CYP2C19* polymorphisms and CAS risk was analyzed. There were 1,403(30.3%) patients with dyslipidemia, and dyslipidemia (41.1% vs. 18.8%, *p* < 0.001) in patients with CAS was higher than those in controls. The frequencies of *CYP2C19* IM and PM phenotypes in CAS patients were higher than those in controls. Logistic regression analysis showed that advanced age (odds ratio (OR): 1.273, 95% confidence interval (CI): 1.099–1.473, *p* = 0.001), overweight (OR: 1.175, 95% CI: 1.039–1.328, *p* = 0.010), smoking (OR: 1.238, 95% CI: 1.049–1.462, *p* = 0.012), alcoholism (OR: 1.445, 95% CI: 1.040–2.007, *p* = 0.028), hypertension (OR: 1.229, 95% CI: 1.047–1.442, *p* = 0.012), dyslipidemia (OR: 3.027, 95% CI: 2.641–3.469, *p* < 0.001), *CYP2C19* IM/PM phenotype(IM + PM vs. EM phenotype, OR: 1.290, 95% CI: 1.140–1.459, *p* < 0.001) were associated with CAS in T2DM. *CYP2C19* IM + PM phenotypes, advanced age, overweight, history of smoking, history of alcoholism, hypertension, and dyslipidemia may be associated with CAS in T2DM.

## Introduction

Coronary atherosclerosis (CAS) is a chronic progressive disease mainly characterized by lipid deposition, inflammatory cell infiltration, fibrous tissue hyperplasia, and atherosclerotic plaque formation in the coronary artery vessel wall (1, 2). As the disease progresses, the coronary artery plaques continuously enlarge and protrude into the vascular lumen, resulting in lumen stenosis and hindering myocardial blood supply (3). When plaques rupture or thrombosis forms, it can trigger acute coronary syndrome (ACS), including severe cardiovascular events such as unstable angina pectoris and myocardial infarction (4). The pathological process not only involves lipid metabolism disorders, but is also closely related to multiple mechanisms such as inflammatory responses, oxidative stress, and vascular endothelial dysfunction (5–7). CAS has become one of the cardiovascular diseases with the highest incidence and mortality rates worldwide (8, 9). Cardiovascular diseases cause approximately 17.9 million deaths each year, among which coronary artery disease (CAD) accounts for the major proportion according to statistical data (10). CAS not only causes patients to suffer from long-term symptoms such as angina pectoris and breathing difficulties, seriously affecting their quality of life (11), but may also trigger complications such as heart failure and arrhythmia (12).

Patients with diabetes mellitus, due to being in a state of high blood glucose for a long time, will trigger a series of metabolic disorders, thereby accelerating the process of CAS (13). High blood glucose can cause damage to vascular endothelial cells, making the originally smooth inner lining of blood vessels rough and creating conditions for lipid deposition (14). Meanwhile, it activates signaling pathways such as protein kinase C (PKC), promotes the release of inflammatory factors, and intensifies the inflammatory response of the vascular wall (15). In addition, high blood glucose can also affect platelet function and increase the risk of thrombosis (16). The risk of cardiovascular disease for diabetic patients is 2–4 times that of non-diabetic patients, among which CAD is one of the leading causes of death for patients with diabetes mellitus (17). Moreover, once patients with diabetes mellitus develop CAS, their condition is often more severe, the prognosis is worse, and the incidence of adverse cardiovascular events such as acute myocardial infarction and heart failure increases significantly (18, 19).

Cytochrome P450 2C19 (CYP2C19), as an important drug-metabolizing enzyme in the human body, its genetic polymorphism can lead to significant differences in enzyme activity and be classified into different metabolic types. The rs4244285 (681G > A, *2) and rs4986893 (636G > A, *3) are the two common single-nucleotide polymorphisms (SNPs) in *CYP2C19* (20, 21). Based on the variants of these two SNPs, there are 6 genotypes for *CYP2C19*: *1/*1, *1/*2, *1/*3, *2/*2, *2/*3, and *3/*3 (22), and 3 phenotypes: extensive metabolizer (EM) (*1/*1), intermediate metabolizer (IM) (*1/*2 and *1/*3), and poor metabolizer (PM) (*2/*2, *2/*3, and *3/*3) (23, 24). CYP2C19 is involved in the metabolic processes of some cardiovascular drugs, such as antiplatelet drugs like clopidogrel (25). CYP2C19 can also affect the pathological process of CAS by participating in endogenous substance metabolism in the body, such as the metabolic regulation of lipids, inflammatory factors, and oxidative stress-related molecules (26). CYP2C19 may be involved in the arachidonic acid metabolic pathway and affect the generation of inflammatory mediators, while the inflammatory response plays a key role in the formation, progression and rupture of atherosclerotic plaques (27, 28). Furthermore, the reactive oxygen species (ROS) generated during the metabolic reactions of coronary endothelial cells involving CYP450 will inhibit the vasodilation mediated by nitric oxide (NO) (29). The relationship between *CYP2C19* gene polymorphisms and the risk of CAS in patients with T2DM remains unclear. This study intends to conduct research on it.

## Materials and methods

### Participants

This study is a case–control study. From January 2019 to March 2024, patients with T2DM were continuously included in the outpatient and inpatient departments of Meizhou People’s Hospital. The case group consists of patients who have both T2DM and CAS simultaneously. Inclusion criteria: (1) meeting the diagnostic criteria for T2DM; (2) patients with CAS; and (3) the clinical data were complete (including medical history, physical examination, laboratory tests, and imaging data). T2DM was defined as blood glucose ≥11.1 mol/L at any time or fasting blood glucose ≥7.0 mol/L, or 2-h postprandial plasma glucose level ≥11.1 mol/L (30). The diagnostic criteria for CAD: coronary angiography (CAG) showed that the degree of lumen stenosis of at least one coronary artery was ≥50% (31). The control group consisted of patients with T2DM only.

**Figure 1:**
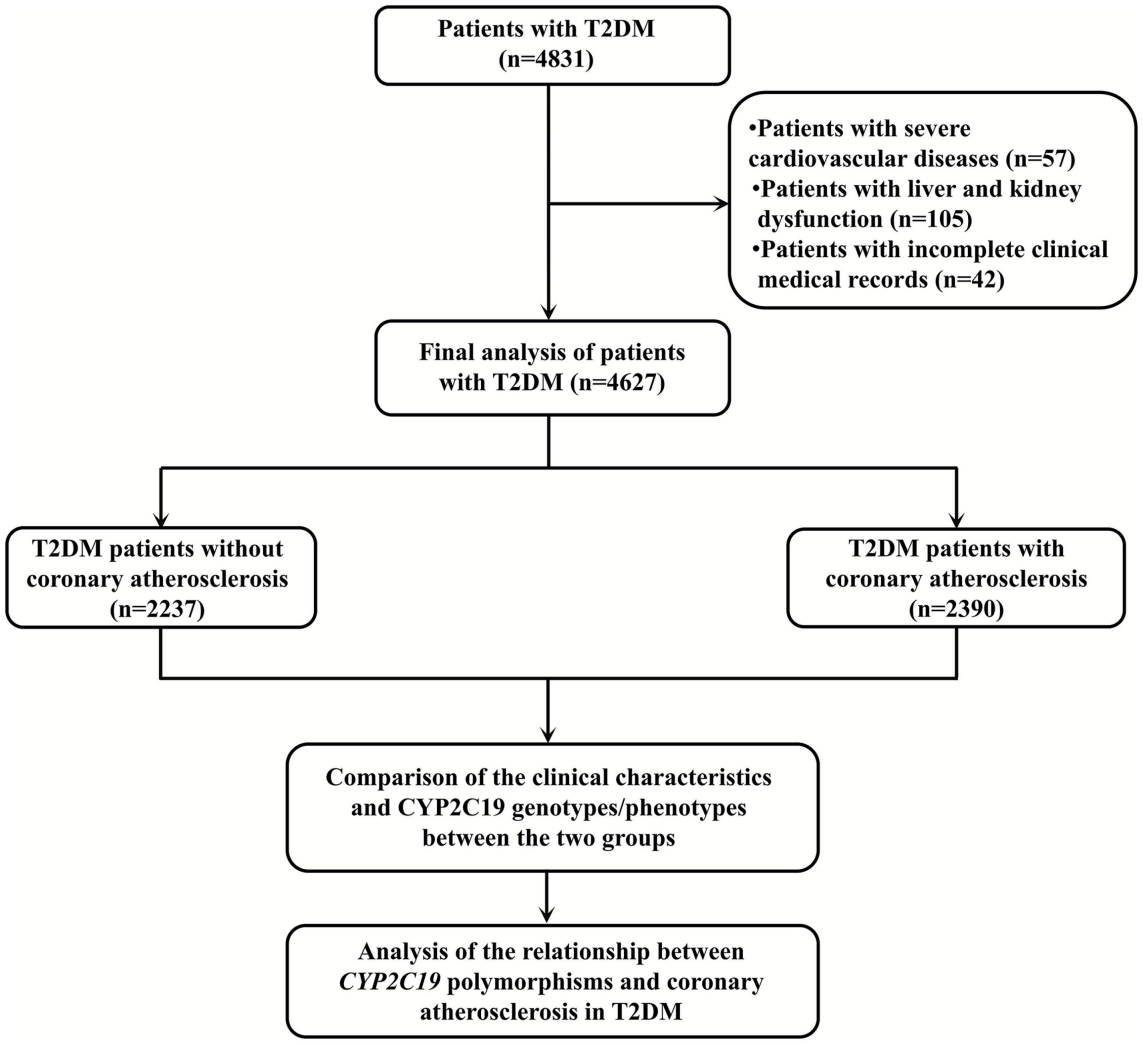
The flowchart of this study.

Exclusion criteria: (1) complicated with malignant tumors, severe liver or kidney dysfunction, autoimmune diseases or hereditary metabolic disorders; (2) had coronary artery bypass surgery or percutaneous coronary intervention in the past; (3) had a major surgical history or severe infection within the last 3 months; (4) clinical data is incomplete. Ultimately, 4,627 patients with T2DM were included in this study, including 2,390 patients with CAS and 2,237 without. This study was supported by the Ethics Committee of the Meizhou People’s Hospital (Clearance No.: 2016-A-55). The flowchart of this study is shown in Figure 1.

### Data collection, definition of indicator grouping, and CYP2C19 gene testing

Clinical data collected including gender, age, body mass index (BMI), history of smoking, history of alcohol consumption, hypertension, and lipid level test results. BMI was divided into three grades: underweight (<18.5 kg/m^2^), normal weight (18.5–23.9 kg/m^2^), and overweight (≥24.0 kg/m^2^) (32, 33). A diagnosis of dyslipidemia can be made if one of the following conditions is met: (1) total cholesterol (TC) ≥ 6.22 mmol/L, (2) triglyceride (TG) ≥ 2.26 mmol/L, or (3) low-density lipoprotein-cholesterol (LDL-C) ≥ 4.14 mmol/L, according to the Guidelines for the Prevention and Control of Dyslipidemia in Chinese Adults (34, 35).

*CYP2C19* genotyping was conducted using previously described method by detecting the genomic DNA of peripheral blood cells from the subjects included in the study (22, 36). Genomic DNA was isolated from peripheral whole blood specimens using the QIAamp DNA Blood Mini Kit (Qiagen GmbH, North Rhine-Westphalia, Germany), following the manufacturer’s standardized operating protocol. Genotyping of the *CYP2C19* *2 and *CYP2C19* *3 variant alleles was implemented with a dedicated *CYP2C19* genotyping kit. Polymerase chain reaction (PCR) amplification was carried out under the following cycling conditions: initial denaturation at 94 °C for 5 min; 35 cycles of amplification consisting of denaturation at 94 °C for 25 s, annealing at 48 °C for 40 s, and extension at 72 °C for 30 s; and a final extension step at 72 °C for 5 min, with the assay system supplied by BaiO Technology Co, Ltd. (Shanghai, China). Post-amplification, the obtained PCR products were hybridized with wild-type and mutant-specific probes that had been pre-immobilized on gene chips, and the genotype of each sample was subsequently determined based on the hybridization signal profiles generated. To guarantee the precision and credibility of experimental data, positive controls, negative controls, and blank controls were included in each batch of tests. The dataset from each experimental batch was considered valid only when all three control types produced consistent and anticipated results.

### Statistical analysis

The statistical analyses of this study were conducted using SPSS 26.0 software (IBM Inc., USA). Continuous variables were compared using either Student’s *t*-test or Mann–Whitney *U* test. The *χ*^2^ test was used to compare the genotypes and alleles frequencies among different groups, and to evaluate the Hardy–Weinberg equilibrium in the patients and controls. Logistic regression analysis was applied to examine the relationship of CYP2C19 phenotypes and CAS susceptibility *p* < 0.05.

## Results

### Characteristics of subjects

A total of 2,873 male patients (62.1%) and 1754 female patients (37.9%) were enrolled in the present study. Of the study cohort, 3,542 individuals (76.6%) were aged 60 years or older, and 2,379 cases (51.4%) were classified as overweight. With regard to medical history, 987 patients (21.3%) had a smoking history, 184 (4.0%) had a history of alcohol consumption, and 3,798 (82.1%) were diagnosed with hypertension, respectively. The median (interquartile range) levels of TC, TG, HDL-C, and LDL-C were 4.52 (3.72, 5.38) mmol/L, 1.61 (1.18, 2.23) mmol/L, 1.13 (0.96, 1.34) mmol/L, and 2.60 (2.02, 3.23) mmol/L, respectively. Additionally, dyslipidemia was identified in 1403 patients, accounting for 30.3% of the total population (Table 1).

**Table 1:** Comparison of clinical features of coronary atherosclerosis patients and non-coronary atherosclerosis controls among patients with T2DM.

Variables	Total (*n* = 4,627)	Controls (*n* = 2,237)	Coronary atherosclerosis patients (*n* = 2,390)	*p* (*χ*^2^)
Gender				
Male, *n* (%)	2,873 (62.1%)	1,407 (62.9%)	1,466 (61.3%)	0.275 (*χ*^2^ = 1.191)
Female, *n* (%)	1,754 (37.9%)	830 (37.1%)	924 (38.7%)	
Age (years)				
<60, *n* (%)	1,085 (23.4%)	543 (24.3%)	542 (22.7%)	0.211 (*χ*^2^ = 1.639)
≥60, *n* (%)	3,542(76.6%)	1,694(75.7%)	1,848(77.3%)	
BMI (kg/m^2^)				
Underweight, *n* (%)	112 (2.4%)	63 (2.8%)	49 (2.1%)	0.001 (*χ*^2^ = 15.048)
Normal weight, *n* (%)	2,136 (46.2%)	1,087 (48.6%)	1,049 (43.9%)	
Overweight, *n* (%)	2,379 (51.4%)	1,087 (48.6%)	1,292 (54.1%)	
History of smoking				
No, *n* (%)	3,640 (78.7%)	1,792 (80.1%)	1,848 (77.3%)	0.022 (*χ*^2^ = 5.341)
Yes, *n* (%)	987 (21.3%)	445 (19.9%)	542 (22.7%)	
History of alcohol consumption				
No, *n* (%)	4,443 (96.0%)	2,166 (96.8%)	2,277 (95.3%)	0.008 (*χ*^2^ = 7.309)
Yes, *n* (%)	184 (4.0%)	71 (3.2%)	113 (4.7%)	
Hypertension				
No, *n* (%)	829 (17.9%)	425 (19.0%)	404 (16.9%)	0.066 (*χ*^2^ = 3.448)
Yes, *n* (%)	3,798 (82.1%)	1,812 (81.0%)	1,986 (83.1%)	
Levels of serum lipid				
TC, mmol/L, median (IQR)	4.52 (3.72, 5.38)	4.40 (3.64, 5.19)	4.63 (3.80, 5.54)	<0.001
TG, mmol/L, median (IQR)	1.61 (1.18, 2.23)	1.48 (1.12, 1.93)	1.79 (1.27, 2.58)	<0.001
HDL-C, mmol/L, median (IQR)	1.13 (0.96, 1.34)	1.16 (0.99, 1.36)	1.10 (0.94, 1.32)	<0.001
LDL-C, mmol/L, median (IQR)	2.60 (2.02, 3.23)	2.62 (2.04, 3.23)	2.58 (1.99, 3.23)	0.430
Dyslipidemia				
No, *n* (%)	3,224 (69.7%)	1,816 (81.2%)	1,408 (58.9%)	<0.001 (*χ*^2^ = 271.190)
Yes, *n* (%)	1,403 (30.3%)	421 (18.8%)	982 (41.1%)	

T2DM, type 2 diabetes mellitus; BMI, body mass index; TC, total cholesterol; TG, triglycerides; HDL-C, high-density lipoprotein-cholesterol; LDL-C, low-density lipoprotein-cholesterol; IQR, interquartile range.

### Comparison of clinical features of CAS patients and non-CAS controls among patients with T2DM

There was significant difference in the distribution of BMI (*χ*^2^ = 15.048, *p* = 0.001) between patients with CAS and controls. The proportion of history of smoking (22.7% vs. 19.90%, *p* = 0.022), and history of alcohol consumption (4.7% vs. 3.2%, *p* = 0.008), and dyslipidemia (41.1% vs. 18.8%, *p* < 0.001) in patients with CAS was higher than those in controls, respectively (Table 2).

**Table 2:** Comparison of the *CYP2C19* genotypes and alleles between patients with coronary atherosclerosis and non-coronary atherosclerosis controls.

CYP2C19 phenotypes	*CYP2C19* genotypes/alleles	Total (*n* = 4,627)	Controls (*n* = 2,237)	Coronary atherosclerosis patients (*n* = 2,390)	*χ* ^2^	*p* values
	Genotypes					
Extensive metabolizer	*1/*1	1,826 (39.5%)	954 (42.6%)	872 (36.5%)	18.359	<0.001
Intermediate metabolizer	*1/*2	1,901 (41.1%)	956 (42.7%)	945 (39.5%)	4.876	0.029
	*1/*3	271 (5.9%)	93 (4.2%)	178 (7.4%)	22.688	<0.001
Poor metabolizer	*2/*2	492 (10.6%)	187 (8.4%)	305 (12.8%)	23.564	<0.001
	*2/*3	127 (2.7%)	44 (2.0%)	83 (3.5%)	9.816	0.002
	*3/*3	10 (0.2%)	3 (0.1%)	7 (0.3%)	1.351	0.346
	Alleles					
	*1	5,824 (62.9%)	2,957 (66.1%)	2,867 (60.0%)	37.032	<0.001
	*2	3,012 (32.5%)	1,374 (30.7%)	1,638 (34.3%)	13.318	<0.001
	*3	418 (4.5%)	143 (3.2%)	275 (5.8%)	35.031	<0.001
	HWE (*χ*^2^, *p*)	*χ*^2^ = 0.921, *p* = 0.922	*χ*^2^ = 6.049, *p* = 0.196	*χ*^2^ = 6.192, *p* = 0.185		

HWE, Hardy Weinberg Equilibrium.

### Comparison of the *CYP2C19* genotypes and alleles between patients with CAS and controls

The genotype distribution of *CYP2C19* in all subjects conforms to the Hardy–Weinberg equilibrium (*χ*^2^ = 0.921, *p* = 0.922). The proportion of cases carried *CYP2C19* *1/*1, *1/*2, *1/*3, *2/*2, *2/*3, and *3/*3 genotype was 39.5, 41.1, 5.9, 10.6, 2.7, and 0.2%, respectively. There were 1826 (39.5%), 2,172 (46.9%), and 629 (13.6%) cases with *CYP2C19* EM, IM, and PM phenotype, respectively. The *CYP2C19* *1 allele frequency was lower (60.0% vs. 66.1%, *p* < 0.001), *CYP2C19* *2 (34.3% vs. 30.7%, *p* < 0.001) and *3 (5.8% vs. 3.2%, *p* < 0.001) allele frequencies in CAS patients were higher than those in controls (Table 2).

### Comparison of clinical characteristics of individuals with different *CYP2C19* phenotypes

There were significant differences in the proportions of individuals with hypertension and dyslipidemia among patients with different CYP2C19 phenotypes. There were no significant differences in the distribution of gender, age, BMI, and the proportions of smoking and drinking history among patients with different CYP2C19 phenotypes (Table 3).

**Table 3:** Comparison of clinical characteristics of cases with different CYP2C19 phenotypes.

Variables	Extensive metabolizer (*n* = 1,826)	Intermediate metabolizer (*n* = 2,172)	Poor metabolizer (*n* = 629)	*p* (*χ*^2^)
Gender				
Male, *n* (%)	1,157 (63.4%)	1,349 (62.1%)	367 (58.3%)	0.082 (*χ*^2^ = 5.001)
Female, *n* (%)	669 (36.6%)	823 (37.9%)	262 (41.7%)	
Age (years)				
<60, *n* (%)	427 (23.4%)	502 (23.1%)	156 (24.8%)	0.676 (*χ*^2^ = 0.782)
≥60, *n* (%)	1,399 (76.6%)	1,670 (76.9%)	473 (75.2%)	
BMI (kg/m^2^)				
Underweight, *n* (%)	40 (2.2%)	61 (2.8%)	11 (1.7%)	0.421 (*χ*^2^ = 3.889)
Normal weight, *n* (%)	837 (45.8%)	996 (45.9%)	303 (48.2%)	
Overweight, *n* (%)	949 (52.0%)	1,115 (51.3%)	315 (50.1%)	
History of smoking				
No, *n* (%)	1,410 (77.2%)	1,720 (79.2%)	510 (81.1%)	0.090 (*χ*^2^ = 4.823)
Yes, *n* (%)	416 (22.8%)	452 (20.8%)	119 (18.9%)	
History of alcohol consumption				
No, *n* (%)	1,750 (95.8%)	2,086 (96.0%)	607 (96.5%)	0.754 (*χ*^2^ = 0.554)
Yes, *n* (%)	76 (4.2%)	86 (4.0%)	22 (3.5%)	
Hypertension				
No, *n* (%)	224 (12.3%)	448 (20.6%)	157 (25.0%)	<0.001 (*χ*^2^ = 71.689)
Yes, *n* (%)	1,602 (87.7%)	1,724 (79.4%)	472 (75.0%)	
Dyslipidemia				
No, *n* (%)	1,317 (72.1%)	1,505 (69.3%)	402 (63.9%)	<0.001 (*χ*^2^ = 15.230)
Yes, *n* (%)	509 (27.9%)	667 (30.7%)	227 (36.1%)	

T2DM, type 2 diabetes mellitus; BMI, body mass index.

### Logistic regression analysis of the relationship of CYP2C19 phenotypes and CAS risk

By univariate analysis, there were significant interactions between CYP2C19 phenotypes and traditional risk factors, such as history of smoking, history of alcoholism, hypertension, and dyslipidemia (Table 4). However, in a multivariate analysis, we found the interaction between CYP2C19 phenotypes and traditional risk factors was not statistically significant. Univariate regression logistic analysis showed that overweight (odds ratio (OR): 1.232, 95% confidence interval (CI): 1.096–1.385, *p* < 0.001), smoking (OR: 1.181, 95% CI: 1.026–1.360, *p* = 0.021), alcoholism (OR: 1.514, 95% CI: 1.119–2.049, *p* = 0.007), dyslipidemia (OR: 3.008, 95% CI: 2.632–3.439, *p* < 0.001), *CYP2C19* IM phenotype (IM vs. EM phenotype, OR: 1.171, 95% CI: 1.034–1.327, *p* = 0.013) and PM phenotype (PM vs. EM phenotype, OR: 1.847, 95% CI: 1.533–2.224, *p* < 0.001) were significantly associated with CAS in T2DM. Multivariate regression logistic analysis showed that advanced age (OR: 1.273, 95% CI: 1.099–1.473, *p* = 0.001), overweight (OR: 1.175, 95% CI: 1.039–1.328, *p* = 0.010), smoking (OR: 1.238, 95% CI: 1.049–1.462, *p* = 0.012), alcoholism (OR: 1.445, 95% CI: 1.040–2.007, *p* = 0.028), hypertension (OR: 1.229, 95% CI: 1.047–1.442, *p* = 0.012), dyslipidemia (OR: 3.027, 95% CI: 2.641–3.469, *p* < 0.001), *CYP2C19* IM + PM phenotype (IM + PM vs. EM phenotype, OR: 1.290, 95% CI: 1.140–1.459, *p* < 0.001) were associated with CAS in T2DM (Table 5).

**Table 4:** Association of traditional risk factors with the CYP2C19 phenotypes on CAS.

Traditional risk factors	CYP2C19 phenotypes	OR (95% CI)	*p* values
History of smoking			
No	Extensive metabolizer	1.000 (reference)	
No	Intermediate metabolizer + Poor metabolizer	1.272 (1.113–1.454)	0.102
Yes	Extensive metabolizer	1.123 (0.903–1.398)	0.297
Yes	Intermediate metabolizer + Poor metabolizer	1.583 (1.301–1.927)	<0.001
History of alcoholism			
No	Extensive metabolizer	1.000 (reference)	
No	Intermediate metabolizer + Poor metabolizer	1.297 (1.150–1.463)	0.091
Yes	Extensive metabolizer	1.531 (0.962–2.437)	0.073
Yes	Intermediate metabolizer + Poor metabolizer	1.970 (1.316–2.950)	0.001
Hypertension			
No	Extensive metabolizer	1.000 (reference)	
No	Intermediate metabolizer + Poor metabolizer	6.948 (4.757–10.146)	<0.001
Yes	Extensive metabolizer	4.970 (3.483–7.094)	<0.001
Yes	Intermediate metabolizer + Poor metabolizer	5.094 (3.583–7.244)	<0.001
Dyslipidemia			
No	Extensive metabolizer	1.000 (reference)	
No	Intermediate metabolizer + Poor metabolizer	1.221 (1.059–1.408)	<0.001
Yes	Extensive metabolizer	2.797 (2.259–3.462)	<0.001
Yes	Intermediate metabolizer + Poor metabolizer	3.805 (3.168–4.570)	<0.001

T2DM, type 2 diabetes mellitus; BMI, body mass index; OR, odds ratio; CI, confidence interval.

**Table 5:** Logistic regression analysis of risk factors for coronary atherosclerosis in T2DM.

Variables	Univariate OR (95% CI)	*p* values	Multivariate OR (95% CI)	*p* values
Gender (Male vs. Female)	1.068 (0.949–1.203)	0.275	1.059 (0.925–1.214)	0.405
Age (≥60 vs. <60 years old)	1.093 (0.954–1.252)	0.201	1.273 (1.099–1.473)	0.001
BMI (kg/m^2^)				
Normal weight	1.000 (reference)	–	1.000 (reference)	–
Underweight	0.806 (0.550–1.182)	0.269	0.856 (0.577–1.270)	0.439
Overweight	1.232 (1.096–1.385)	<0.001	1.175 (1.039–1.328)	0.010
History of smoking (Yes vs. No)	1.181 (1.026–1.360)	0.021	1.238 (1.049–1.462)	0.012
History of alcoholism (Yes vs. No)	1.514 (1.119–2.049)	0.007	1.445 (1.040–2.007)	0.028
Hypertension (Yes vs. No)	1.153 (0.992–1.340)	0.063	1.229 (1.047–1.442)	0.012
Dyslipidemia (Yes vs. No)	3.008 (2.632–3.439)	<0.001	3.027 (2.641–3.469)	<0.001
*CYP2C19* phenotypes				
Extensive metabolizer	1.000 (reference)	–	1.000 (reference)	–
Intermediate metabolizer	1.171 (1.034–1.327)	0.013	1.172 (1.030–1.334)	0.016
Poor metabolizer	1.847 (1.533–2.224)	<0.001	1.827 (1.505–2.216)	<0.001
Intermediate metabolizer + Poor metabolizer	1.294 (1.150–1.457)	<0.001	1.290 (1.140–1.459)	<0.001

T2DM, type 2 diabetes mellitus; BMI, body mass index; OR, odds ratio; CI, confidence interval.

## Discussion

The clinical management of diabetes mellitus complicated with CAS remains challenging (37). Moreover, the more severe the degree of diabetes mellitus, the higher the probability of cardiovascular events in patients, and the corresponding risk also increases (38). Identifying patients at high risk of CAS in diabetes mellitus patients is the key to prevention. In this study, *CYP2C19* IM + PM phenotype, advanced age, overweight, history of smoking, history of alcoholism, hypertension, and dyslipidemia were associated with CAS risk in T2DM.

Polymorphisms in the *CYP2C19* gene may display substantial racial variations (39–42). In the present study, the proportions of CYP2C19 EM, IM, and PM phenotypes were 39.5, 46.9, and 13.6%, respectively, which are consistent with the findings of several prior studies (43–45). Park et al.’s (46) study on a group of patients undergoing percutaneous coronary intervention (PCI) from East Asia revealed that the proportions of CYP2C19 EM, IM, and PM were 39.8, 45.8, and 14.3%, respectively. A study on the genetic polymorphisms of CYP2C19 in the Chinese Hakka population revealed that the proportions of CYP2C19 EM, IM, and PM were 41.73, 45.21, and 13.06%, respectively (47). Another study conducted among the Hakka population in China revealed that CYP2C19 EM, IM, and PM were 40.8, 46.9, and 12.4%, respectively (22). The distributions of CYP2C19 genotypes reported in most studies of the Asian population is largely similar (43). In addition, CYP2C19 IM and PM statuses were significantly associated with CAS in patients with T2DM in this study. To our knowledge, relatively few investigations have explored the association between *CYP2C19* gene polymorphisms and the risk of CAS. For instance, Chen et al. (48) demonstrated that gene polymorphisms leading to CYP2C19 dysfunction constitute a risk factor for premature coronary artery disease (PCAD). Additionally, Hokimoto et al. (49) revealed that CYP2C19 PM status might be a risk factor for CAD in female populations. Han et al. (50) further found that CYP2C19 PM status is an independent risk factor for premature myocardial infarction (PMI).

Dyslipidemia plays a key role in the process of CAS in patients with T2DM. In patients with T2DM, the hyperglycemic state can promote the glycation modification of LDL-C particles, and the glycated LDL is more likely to be oxidized to form oxidized LDL (ox-LDL) (51). ox-LDL is highly cytotoxic and can induce vascular endothelial cells to express adhesion molecules such as intercellular adhesion molecule-1 (ICAM-1) and vascular cell adhesion molecule-1 (VCAM-1), promoting the adhesion of monocytes to the vascular endothelium and their migration to the vascular intima (52). Monocytes entering the intima take up ox-LDL and transform into foam cells. A large number of foam cells accumulate to form a lipid core, accelerating the formation of atherosclerotic plaques (53). Elevated triglyceride (TG) level also has significant effects in T2DM complicated with CAS. Lipoprotein residues rich in TG accumulate in the body. These residues are easily taken up by macrophages and promote the formation of foam cells (54). Meanwhile, there is lipid exchange between TG and HDL-C as well as LDL-C. An increase in TG will cause the cholesterol esters in HDL-C to transfer to lipoproteins rich in TG, resulting in smaller HDL-C particles and reduced quantity, weakening its mediated reverse cholesterol transport function, and preventing cholesterol from being effectively transported from the vascular wall to the liver for excretion from the body (55). In addition, insulin resistance is widespread among patients with T2DM. Insulin resistance prompts the liver to synthesize more very low-density lipoprotein (VLDL), causing dyslipidemia, especially elevated triglycerides and decreased high-density lipoprotein cholesterol (HDL-C) (56). Both of these dyslipidemia are important risk factors for CAS.

In addition, hypertension and T2DM often coexist (57). The hemodynamic changes caused by hypertension exert mechanical stress on the vascular wall, disrupt the integrity of the vascular endothelium, increase the permeability of vascular endothelial cells, promote lipid infiltration and the aggregation of inflammatory cells, and together with diabetes mellitus, significantly increase the risk of CAS (58). Overweight cannot be ignored in the development of CAS in patients with T2DM. Adipose tissue, as an important endocrine organ, is characterized by excessive proliferation and hypertrophy of adipocytes in patients with central obesity, which secretes a large amount of free fatty acids, inflammatory factors (such as tumor necrosis factor -*α*, interleukin-6), and adipokines (such as decreased adiponectin levels and elevated resistin levels) (59). Free fatty acids can interfere with insulin signal transduction and aggravate insulin resistance. Inflammatory factors can activate the inflammatory response of vascular endothelial cells and promote the adhesion and migration of monocytes (60). These factors interweave with each other, creating conditions for the occurrence of CAS.

To our knowledge, this study is one of the few that have reported the relationship between *CYP2C19* gene polymorphism and the risk of CAS in patients with T2DM. Although this study has identified some risk factors for coronary atherosclerosis in patients with T2DM, there are still some deficiencies. Firstly, in this retrospective study, only common risk factors were included, and some rare and potential factors were not investigated. Secondly, without data on whether patients with T2DM complicated with hypertension received antihypertensive treatment, it is impossible to study the relationship between post-treatment blood pressure and the risk of CAS. Thirdly, this study lacks information regarding the medication history of the enrolled patients. Detailed data on the medications used by the subjects both before and after disease onset were not collected. Consequently, we cannot rule out the confounding effect of pharmacotherapy on the association analysis between *CYP2C19* genotypes and CAS risk, nor can we clarify whether pharmacotherapy modifies the impact of genotypes on the incidence risk of CAS. Finally, our study is observational in design and cannot establish a direct causal link between CYP2C19 metabolizer status and CAS risk in T2DM patients; additionally, our findings are specific to the T2DM cohort, and caution against extrapolating this role of CYP2C19 to non-T2DM populations. Future prospective cohort studies or randomized controlled trials are warranted to validate the modulatory role of CYP2C19 genotype in this context.

CAS, as a preventable disease, the identification of high-risk patients is the key to the prevention and control of the disease. In future research, several aspects deserve attention. First, there may be different pathogenic factors in the occurrence process of CAS. What are the interaction patterns and mechanisms of these pathogenic factors? Second, there are many risk factors for cardiovascular diseases, including CAS. Is it genetic or external factor that plays a dominant role? Which risk factors can be effectively controlled to reduce the incidence of cardiovascular diseases?

## Conclusion

This study investigated the association between *CYP2C19* gene polymorphism and CAS in patients with T2DM. The results indicated that the intermediate metabolizer and poor metabolizer phenotypes of CYP2C19 may be correlated with CAS in T2DM patients. This finding provides a novel perspective for exploring the pathogenesis of CAS in T2DM patients, as well as potential targets and a reference basis for precise prevention and treatment. However, this study has limitations including a small sample size and the lack of clarification regarding specific molecular mechanisms. Future research should conduct multicenter, large-sample prospective studies and basic experiments for further verification and investigation. The ultimate goal is to translate the CYP2C19 metabolic phenotype into a biomarker for CAS risk assessment in T2DM patients, thereby facilitating the development of individualized prevention and treatment strategies.

## Data Availability

The original contributions presented in the study are included in the article/supplementary material, further inquiries can be directed to the corresponding author.
